# Genomic and phenotypic characterization of antimicrobial resistance in clinical *Nocardia* species isolates

**DOI:** 10.3389/fcimb.2025.1672889

**Published:** 2025-09-18

**Authors:** Ziyu Song, Bingqian Du, Min Yuan, Jirao Shen, Shuai Xu, Wanchun Guan, Binghuai Lu, Zhenjun Li

**Affiliations:** ^1^ School of Laboratory Medicine and Life Sciences, Wenzhou Medical University, Wenzhou, China; ^2^ State Key Laboratory of Infectious Disease Prevention and Control, National Institute for Communicable Disease Control and Prevention, Chinese Center for Disease Control and Prevention, Beijing, China; ^3^ Laboratory of Clinical Microbiology and Infectious Diseases, Department of Pulmonary and Critical Care Medicine, Beijing Key Laboratory of Surveillance, Early Warning and Pathogen Research on Emerging Infectious Diseases, National Center for Respiratory Medicine, China-Japan Friendship Hospital, Beijing, China

**Keywords:** Nocardia, whole genome sequencing, antimicrobial resistance, average nucleotide identity, minimum inhibitory concentration

## Abstract

**Background:**

Antimicrobial resistance is prevalent across *Nocardia* species, with varying resistance profiles among different species, which poses significant challenges to effective treatment strategies. Our study aimed to assess the antimicrobial susceptibility profiles of various *Nocardia* species and investigate the potential correlation between resistance phenotypes and their underlying genotypes.

**Methods:**

This study analyzed 148 clinical *Nocardia* isolates from 13 provinces in China. Minimum inhibitory concentrations (MICs) for 15 antimicrobial agents were determined using the microbroth dilution method. Whole genome sequencing (WGS) was performed for all isolates, followed by bioinformatics analyses integrated with 70 human-sourced *Nocardia* genomes in the National Center for Biotechnology Information (NCBI), encompassing species verification, phylogenetic analysis, and the identification of antimicrobial resistance genes (ARGs).

**Results:**

Average Nucleotide Identity (ANI) analysis reclassified several misidentified isolates and revealed 14 potentially novel *Nocardia* species, underscoring the taxonomic complexity within this genus. *Nocardia* species exhibited distinct resistance profiles: *Nocardia farcinica* demonstrated elevated resistance to cephalosporins and tobramycin; *Nocardia otitidiscaviarum* showed broad resistance to *β*-lactams and quinolones; and *Nocardia cyriacigeorgica* exhibited resistance to quinolones, cefepime, and cefoxitin. Notably, clarithromycin resistance was consistently high across all species. Moreover, 38.51% of *Nocardia* isolates are resistant to two or more commonly used antibiotics, which revealed widespread multidrug resistance (MDR). Strong genotype–phenotype correlations were observed, including *sul1* in sulfamethoxazole/trimethoprim-resistant *N. farcinica*, *bla*_AST-1_ in *β*-lactam-resistant *N. otitidiscaviarum*, and *tetA/B(58)* across tetracycline-intermediate isolates. Additionally, resistance mechanisms beyond ARGs were observed, including species-specific presence of *warA* and *aph(2’’)*, and *gyrA* mutations largely correlating with ciprofloxacin resistance. Nonetheless, resistance in some strains lacking known resistance determinants indicates the presence of uncharacterized mechanisms.

**Conclusions:**

These findings provide critical insights into the drug resistance patterns of *Nocardia* strains and antimicrobial resistance genes, highlighting the importance of ongoing genomic surveillance and personalized treatments.

## 1 Introduction

*Nocardia*, a member of the Actinobacteria order, primarily infects immunocompromised individuals, such as those with Human Immunodeficiency Virus (HIV), organ transplants, or chronic lung disease, but can also cause infections in immunocompetent individuals (Shen et al., 2025). If *Nocardia* infection at the primary site is not promptly and effectively treated, it may spread to other parts of the body, causing severe infections and potentially leading to death (Fatahi-Bafghi, 2018). In recent years, with the increasing number of elderly individuals, immunocompromised patients, organ transplant recipients, and HIV-infected patients, the incidence of *Nocardia* infections has been gradually rising (Ji et al., 2022; Shen et al., 2025). Studies suggest that the mortality rate for *Nocardia* bacteremia patients is approximately 50% (Kontoyiannis et al., 1998), while the mortality rate for patients with disseminated infection ranges from 44% (Beaman and Beaman, 1994) to 85% (Presant et al., 1973). As a life-threatening infection, *Nocardia* infection poses significant clinical challenges. However, given the extensive diversity within the *Nocardia* genus, identification of rare species by Matrix-Assisted Laser Desorption/Ionization Time-of-Flight Mass Spectrometry (MALDI-TOF MS) remains challenging for clinical laboratories, despite some species being readily identifiable (Conville et al., 2017). Furthermore, varying drug susceptibility patterns among *Nocardia* species may result in treatment failures (Zhao et al., 2017; Wang et al., 2022; Hershko et al., 2023b). Accurate species identification is essential for understanding the epidemiology and pathogenicity of *Nocardia*. The global rise in antimicrobial resistance (AMR) represents a significant public health threat (GBD 2021 Antimicrobial Resistance Collaborators, 2024). While most *Nocardia* strains remain sensitive to amikacin, linezolid, and trimethoprim-sulfamethoxazole, their susceptibility profiles vary when it comes to *β*-lactam antibiotics, quinolones, aminoglycosides, and other antibiotics (Zhao et al., 2017; Wang et al., 2022; Hershko et al., 2023b).

Reports on the antibiotic sensitivity of *Nocardia* species in various regions of China have been published (Wang et al., 2022; Yi et al., 2019; Wei et al., 2017; Han et al., 2024), but these reports have been limited to the phenotypic aspects of drug resistance and have not explored the genetic basis of drug resistance.

Whole-genome sequencing (WGS), a high-throughput sequencing technology, offers a comprehensive approach to detecting AMR genes in clinical bacterial strains (Balloux et al., 2018). Additionally, WGS facilitates the prediction of bacterial genetic profiles and functional phenotypes (Balloux et al., 2018). Consequently, the application of this technology significantly enhances the efficiency of clinical microbiological testing and provides critical information for species identification of *Nocardia* and guiding therapeutic interventions.

Understanding genetic diversity of *Nocardia* isolates and their resistance mechanisms is essential for informing empirical antibiotic therapy. This study aimed to perform antimicrobial susceptibility testing and WGS on 148 clinical *Nocardia* isolates collected from 13 provinces in China, to assess their drug resistant phenotype, AMR genes, and genetic characteristics. The findings will deepen our understanding of the genetic factors driving resistance in *Nocardia*, as well as the relationship between genetic background and drug resistance. This valuable information will contribute to more effective clinical management of nocardiosis.

## 2 Materials and methods

### 2.1 Strains collection and initial identification

*Nocardia* spp. strains in this study were isolated from clinical specimens collected across 13 provincial-level administrative divisions in China, including provinces, autonomous regions, and municipalities (Beijing, Gansu Province, Guangdong Province, Guangxi Zhuang Autonomous Region, Hebei Province, Henan Province, Heilongjiang Province, Hunan Province, Inner Mongolia Autonomous Region, Ningxia Hui Autonomous Region, Shandong Province, Zhejiang Province, Jiangsu Province). After strain purification, bacterial colonies were cultured on Brain Heart Infusion (BHI) blood agar for at least 48 hours. Freshly picked colonies were then subjected to MALDI-TOF MS (Zybio EXS3000, Zybio Inc., Chongqing, China) analysis using the formic acid/ethanol extraction method.

### 2.2 Antibiotic susceptibility testing

Minimum inhibitory concentration (MIC) was determined using the Sensititre RAPMYCO microdilution panel (Thermo Fisher Scientific, Sunnyvale, CA, USA) according to the manufacturer’s protocol, with results visualized using the R package ggplot2. Additionally, MIC_50_ and MIC_90_ were calculated using R Studio software, version 4.3.3. The AST plate included 15 antibiotics: amikacin, amoxicillin/clavulanic acid, cefepime, cefoxitin, ceftriaxone, ciprofloxacin, clarithromycin, doxycycline, imipenem, linezolid, minocycline, moxifloxacin, tigecycline, tobramycin, trimethoprim/sulfamethoxazole. Quality control was performed using *Staphylococcus aureus* ATCC 29213 and *Escherichia coli* ATCC 25922 as reference strains. Results were interpreted according to the *Nocardia* spp. interpretive criteria specified in Clinical and Laboratory Standards Institute (CLSI) standard M24-A2 (Woods et al., 2011). As the MIC for sulfamethoxazole/trimethoprim is defined as the lowest concentration inhibiting 80% of growth, additional verification was conducted using the MIC test strip (MTS) (Liofilchem Ltd., Roseto Degli Abruzzi, TE, Italy) method to ensure the accuracy of AST results.

### 2.3 DNA extraction and WGS

The strains were subcultured for two consecutive generations in BHI liquid medium. Bacterial cells were then collected by centrifugation to form a pellet. Genomic DNA was extracted from the pellet using the Wizard genomic DNA purification kit (Promega, Madison, WI, USA). Following extraction, DNA purity and concentration were measured using a NanoDrop instrument (Thermo Fisher Scientific, Sunnyvale, CA, USA). Then, the DNA samples were sequenced using the Illumina Novaseq platform in the PE150 mode (Shanghai Majorbio Bio-pharm Technology Co., Ltd., Shanghai, China), ensuring all sequencing depths exceeded 100-fold. The quality of reads was checked and trimmed by Fastp v0.23.4 (Chen, 2023). The cleaned reads were *de novo* assembled by SPAdes v3.15.5 (Bankevich et al., 2012), and the draft genomes were annotated by Prokka v1.12 (Seemann, 2014). The genome size, number of the coding sequences (CDSs), and GC content were estimated by Quast v5.0.2 (Gurevich et al., 2013) and CheckM v1.1.3 (Parks et al., 2015).

### 2.4 Phylogenetic tree construction and average nucleotide identity analysis

To gain a more comprehensive understanding of the classification and genetic evolution of *Nocardia* strains, we downloaded the whole genome sequences of 70 *Nocardia* strains isolated from human origin from the National Center for Biotechnology Information (NCBI) (https://www.ncbi.nlm.nih.gov/datasets/genome/). Pangenome and core genome analysis was conducted using Roary v3.12.0 (Page et al., 2015). Single-copy core genes were identified by BLASTn. Then, the phylogenetic tree based on the core genome was constructed using FastTree and visualized by iTOL (https://itol.embl.de/). Pairwise ANI values across all genomes were calculated by Pyani v0.2.12 (Pritchard et al., 2016) and visualized by the R package pheatmap. The in silico DNA-DNA hybridization (isDDH) values were calculated through the Genome-to-Genome Distance Calculator 2.1 (GGDC) (http://ggdc.dsmz.de/ggdc.php) using “Formula 2” (Auch et al., 2010).

### 2.5 Antibiotic resistance gene analysis

The ARGs were identified using Rgi v6.0.2 with a cutoff of 60% identity and 70% coverage against the Comprehensive Antibiotic Resistance Database (CARD) (https://card.mcmaster.ca/analyze/rgi). AGRs was visualized with the R package pheatmap. Gene presence/absence was analyzed using BLASTn, gene mutations were analyzed via sequence alignment with Clustal Omega (http://lilab2.sysu.edu.cn/Tools/msa/clustalo/). The associations between phenotypic resistance and resistance genes were assessed using Cramér’s V statistic, a measure of association between two categorical variables, ranging from 0 (no association) to 1 (perfect association) (^*^ *P* < 0.05, ^**^ *P* < 0.01, ^***^ *P* < 0.001). The results were visualized as a heatmap, where each cell contains the Cramér’s V value along with its statistical significance. The color intensity reflects the strength of association: yellow shades indicate weak or no association (values close to 0), while deep purple shades indicate strong association (values close to 1).

## 3 Results

### 3.1 Distribution of *Nocardia* species

Among the 148 clinical isolates preliminarily identified by MALDI-TOF MS (**Supplementary Table S1**), 10 *Nocardia* species were characterized, most of which were isolated from the respiratory tract. Specifically, the predominant species were *N. farcinica* (60.8%, 90/148), followed by *N. cyriacigeorgica* (16.2%, 24/148), *N. otitidiscaviarum* (10.8%, 16/148), and *N. wallacei* (5.4%, 8/148). In contrast, less frequently isolated species included *N. brasiliensis* (2.0%, 3/148), *N. nova* (1.4%, 2/148), *N. blacklockiae* (1.4%, 2/148), *N. transvalensis* (0.7%, 1/148), *N. beijingensis* (0.7%, 1/148), and *N. aobensis* (0.7%, 1/148). Notably, *N. farcinica* accounted for over half of all isolates.

### 3.2 Verification of species by ANI

**Figure 1:**
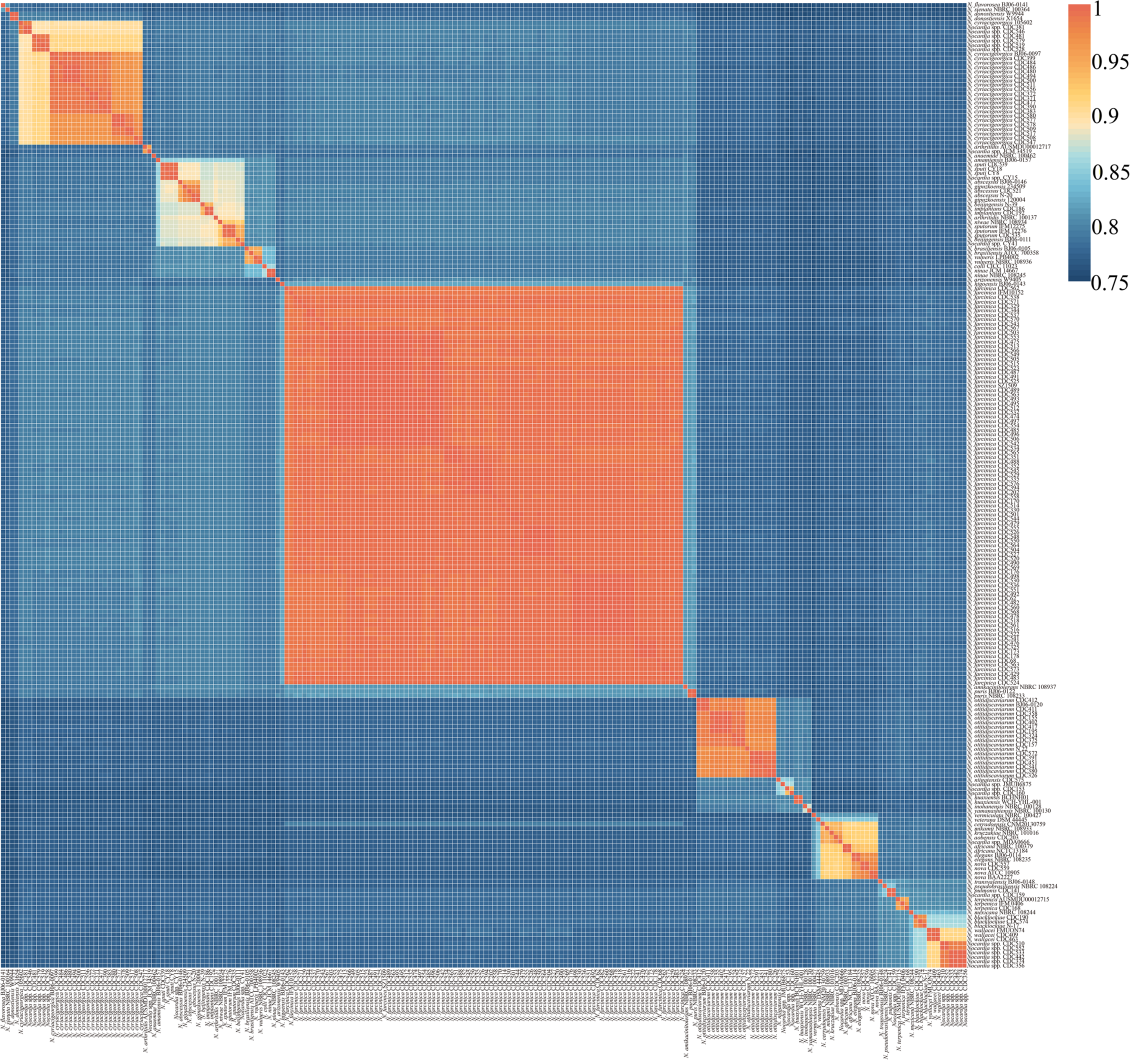
Heatmap of pairwise average nucleotide identity (ANI) values for 218 genome assemblies of *Nocardia* (148 of this study, 70 of NCBI).

A 95-96% ANI threshold was applied to define species boundaries between pairwise genomes (Richter and Rosselló-Móra, 2009). A total of 218 *Nocardia* strains, 148 clinical isolates from this study and 70 clinically isolated strains (comprising 42 species) retrieved from NCBI, were subjected to pairwise ANI comparison. Based on ANI analysis (**Figure 1** and **Supplementary Table S1**), four species without a type strain in NCBI were reclassified (**Supplementary Table S2**), and four *Nocardia* strains from this study were reclassified: *N. brasiliensis* was reclassified as *N. cyriacigeorgica* in two cases; *N. farcinica* as *N.* sp*utorum* in one case; and *N. beijingensis* as *N.* sp*uti* in one case. Additionally, 14 isolates potentially represented taxonomically unclassified species (i.e. novel species) within the genus *Nocardia* (**Supplementary Table S3**). These strains, initially identified by MALDI-TOF MS to *N. cyriacigeorgica* (n=4), *N. wallacei* (n=6), *N. farcinica* (n=1) and *N. blacklockiae* (n=1), exhibited genomic divergence exceeding established species thresholds. Additionally, *N. cyriacigeorgica* 105602, CDC381, CDC481, CDC519, CDC528, CDC546, and CDC579 exhibited the closest ANI with *N. cyriacigeorgica*. However, their ANI values with other *N. cyriacigeorgica* strains were below the 95% species delineation threshold. Consequently, these strains were identified as representing novel/unidentified *Nocardia* species. Phylogenetic analysis further revealed that strains 105602, CDC381, and CDC546 constitute one distinct species, while strains CDC481, CDC519, CDC528, and CDC579 constitute another. Furthermore, six unidentified *Nocardia* strains demonstrated the closest ANI with *N. wallacei*. However, as their ANI values fell below the 95% species delineation threshold, they likely represent novel species phylogenetically related to *N. wallacei*. MALDI-TOF MS correctly identified the majority of *Nocardia* isolates. Except for the unidentified *Nocardia* species, the technique achieved a species-level identification accuracy of 97.1% (132/136) for the isolated strains. This high accuracy provides reliable guidance for the initial clinical screening of *Nocardia* infections.

### 3.3 Genomic characteristics and whole-genome phylogenetic analysis

The genomic characteristics of 148 clinical *Nocardia* isolates and 70 reference strains from NCBI are detailed in **Supplementary Table S4**. The predominant species, *N. farcinica*, *N. otitidiscaviarum*, and *N. cyriacigeorgica*, exhibited mean genome sizes of 6.36 Mb, 7.52 Mb, and 6.44 Mb, respectively, with mean coding sequences (CDSs) of 5,956, 6,892 and 5,820, and mean GC contents of 70.7%, 69.0%, and 68.3%, respectively (**Supplementary Figure S1**).

**Figure 2:**
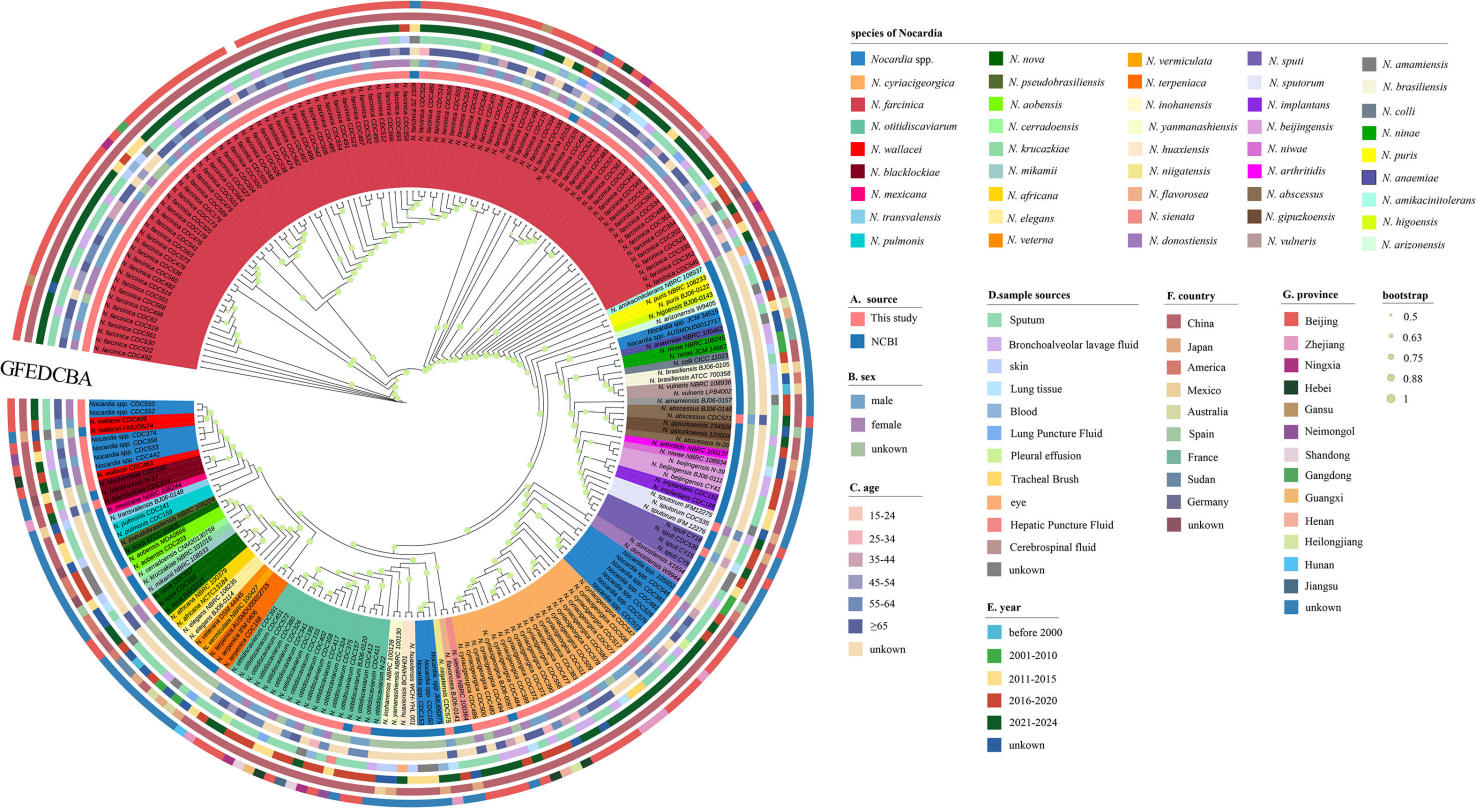
*Nocardia* genome phylogenetic relationships based on the concatenation of thenucleotide sequences of 668 single-copy core genes. The innermost colored bands represent 46 different species of *Nocardia* and *Nocardia* spp. (unable to identify species). **(A)** A colored ring showing different sources of genomic information of *Nocardia*. **(B)** A colored ring showing the gender of patients with *Nocardia* infections. **(C)** A colored ring showing the age of patients with *Nocardia* infections. **(D)** A colored ring showing sample sources of *Nocardia* isolates. **(E)** A colored ring showing five different periods of collection years and the unknown collection years. **(F)** A colored ring showing different country origins of *Nocardia*. **(G)** A colored ring showing different province origins of *Nocardia*.

To further assess the taxonomic relationship among *Nocardia* strains, a circular whole-genome phylogenetic tree was constructed using the whole genome sequence of 668 single-copy genes from 218 *Nocardia* strains (**Figure 2**). The phylogenomic tree, encompassing 46 species, revealed that most strains of the same *Nocardia* species clustered within the same clade. The 148 strains in this study and the 70 strains sourced from NCBI were all isolated from clinical patients. Among the 12 unidentified *Nocardia* strains in this study, 6 exhibited close phylogenetic relationships with *N. cyriacigeorgica*, while the remaining 6 clustered within the *N. wallacei* clade and likely represent novel species within the *N. transvalensis* complex (Conville et al., 2008).

### 3.4 Phenotypic analysis of drug resistance

**Figure 3:**
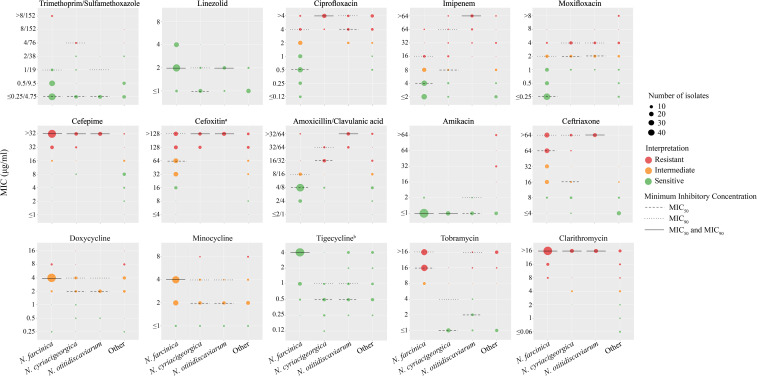
The MIC Minimum Inhibitory Concentrations (MICs) of 15 antimicrobials for *Nocardia* species. The red, orange and green dots indicate the resistant, intermediate and susceptible breakpoints. The size of the points represents the corresponding number of strains. The dashed line represents MIC_50_, the dotted line represents MIC_90_, and the solid black line indicates where MIC_50_ and MIC_90_ coincide. a:The breakpoint for cefotaxime is referenced from the breakpoint for rapidly growing mycobacteria in CLSI-M24-A2. b: The clinical breakpoint for tigecycline is not provided in CLSI M24-A2.

**Figure 3** presents the MIC distributions for 15 AST against the three most prevalent *Nocardia* species in this study, including the MIC_50_, MIC_90_, and resistance rates, AST results for other *Nocardia* species can be found in **Supplementary Table S5**. AST results for sulfamethoxazole/trimethoprim was performed using the MTS method to ensure the accuracy (**Supplementary Table S6**). All clinically isolated *Nocardia* strains demonstrated 100% susceptibility to linezolid, followed by trimethoprim/sulfamethoxazole (140/148, 94.6%) and amikacin (138/148, 93.2%). In contrast, susceptibility to clarithromycin was markedly lower at 4.7% (7/148). For trimethoprim/sulfamethoxazole, MIC values exceeding 8/152 mg/L were exclusively observed in *N. farcinica*. Furthermore, amikacin resistance was detected only in the *N. transvalensis* complex, including *N. wallacei*, *N. blacklockiae*, and some novel/unidentified closely related species of *N. wallacei*.

For *β*-lactam antibiotics, including imipenem, cefepime, cefoxitin, amoxicillin/clavulanic acid, and ceftriaxone, *Nocardia* species exhibited low susceptibility to cephalosporins: cefepime (14.2%), cefoxitin (8.8%), and ceftriaxone (23.0%). Significant interspecies variations in antimicrobial susceptibility were noted. Imipenem susceptibility were 89.8% for *N. farcinica*, compared to 35.0% for *N. cyriacigeorgica* and 0% for *N. otitidiscaviarum*. Similarly, amoxicillin/clavulanic susceptibility varied markedly, with *N. farcinica* at 85.2%, while *N. cyriacigeorgica* and *N. otitidiscaviarum* showed 20.0% and 0% susceptibility, respectively. Moreover, *N. otitidiscaviarum* strains were completely resistance to all *β*-lactam antibiotics, with correspondingly high MIC values.

For tetracyclines, doxycycline and minocycline exhibited high intermediate susceptibility rates of 83.1% and 87.8%. *Nocardia* species displayed distinct susceptibility profiles to quinolone antibiotics, ciprofloxacin and moxifloxacin. *N. farcinica* demonstrated susceptibility rates of 69.3% to ciprofloxacin and 84.1% to moxifloxacin, whereas *N. cyriacigeorgica* and *N. otitidiscaviarum* were completely resistance to ciprofloxacin (100% non-susceptibility) but showed 15.0% and 12.5% susceptibility to moxifloxacin, respectively. Except for *N. farcinica*, other *Nocardia* species have lower MIC values for tigecycline. Tobramycin susceptibility varied significantly: *N. farcinica* exhibited complete resistance (100% non-susceptibility), whereas *N. cyriacigeorgica* and *N. otitidiscaviarum* demonstrated susceptibility rates of 90.0% and 68.8%, respectively.

In this study, we followed the definition proposed by Schlaberg et al (Schlaberg et al., 2014), defining multidrug resistance (MDR) *Nocardia* as isolates that are resistant or intermediate resistant to two or more of the most commonly used empirical antibiotics (amikacin, ceftriaxone, imipenem, and trimethoprim/sulfamethoxazole). The overall MDR rate for clinical *Nocardia* isolates in this study was 38.51% (57/148), with 29.55% (26/88) for *N. farcinica*, 45% (9/20) for *N. cyriacigeorgica*, 100% (16/16) for *N. otitidiscaviarum*, and 100% (2/2) for *N. wallacei*.

### 3.5 Antibiotic resistance genes

**Figure 4:**
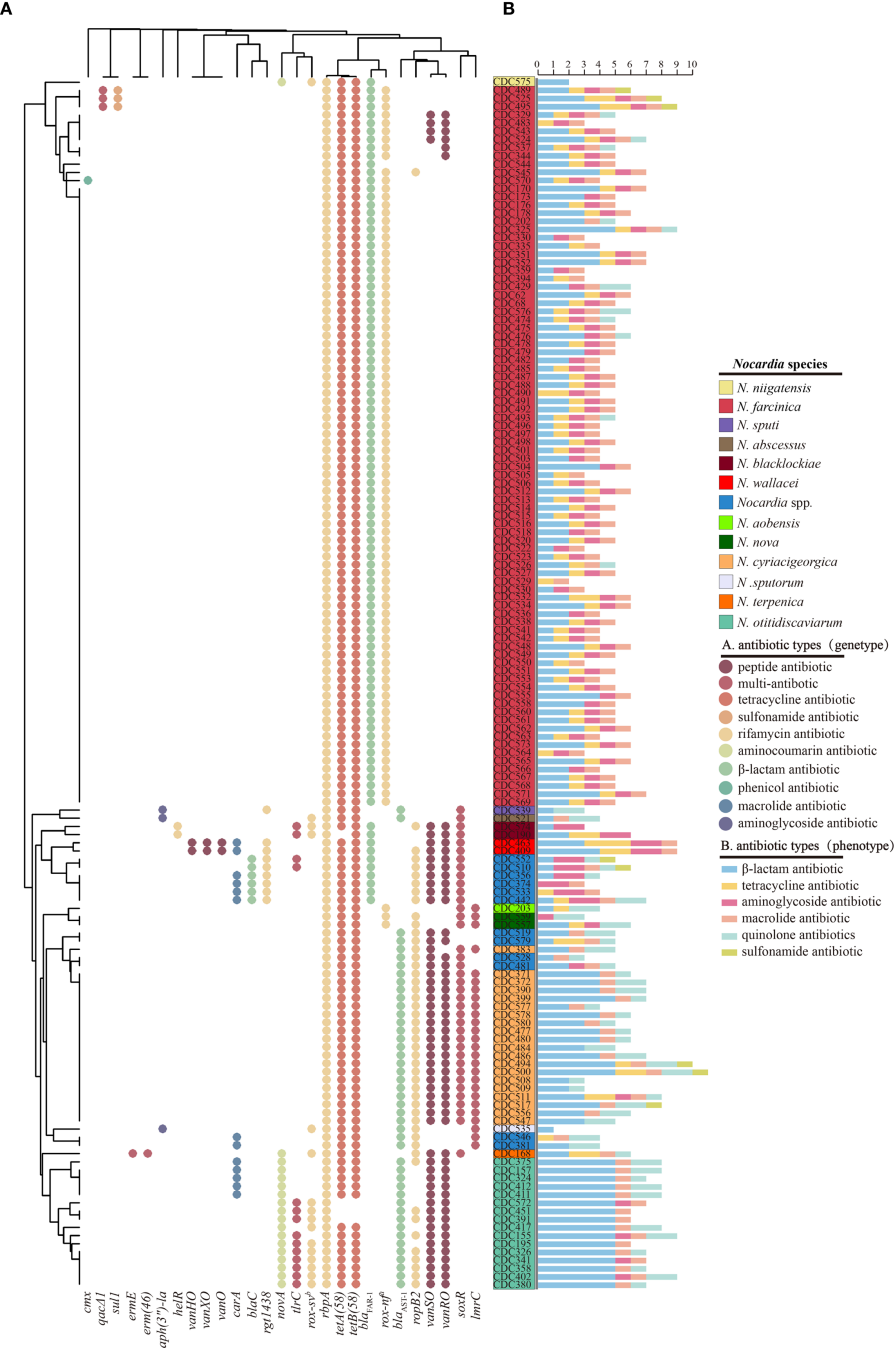
Distribution of Antibiotics Resistance Genes (ARGs) in the genomes of 148 multiple *Nocardia* strains. Each dot indicates the presence of a gene. Different colors indicate different types of ARGs. rox-nf a: *Nocardia farcinica* rox; rox-sv b: *Streptomyces venezuelae* rox **(A)**. Number of antibiotic-resistant phenotypes exhibited by *Nocardia* spp. across six antimicrobial categories **(B)**.

Antibiotic resistance profiling based on WGS of 148 clinical *Nocardia* isolates was visualized through a dot plot (**Figure 4A**), 27 ARGs were identified and these ARGs are involved at least 9 antimicrobial classes of drugs. The rifampicin resistance gene *rbpA* and the tetracycline resistance genes *tetA(58)* and *tetB(58)* were universally present across *Nocardia* species. Additionally, the *β*-lactamase gene *bla*_FAR-1_, conferring partial *β*-lactams antibiotics resistance, predominated in *N. farcinica*, while *vanRO* and *vanSO*, conferring vancomycin resistance, were absent in this species (**Figure 4A**). *bla*_AST-1_ demonstrated species-specific distribution, prevalent in *N. cyriacigeorgica* and *N. otitidiscaviarum*, with its homolog in *N. otitidiscaviarum* linked to pan-*β*-lactam resistance (**Figure 4B**). The lincosamide antibiotic resistance gene *lmrC* was exclusive present in *N. cyriacigeorgica*, while the aminocoumarin antibiotic resistance gene *novA* was unique to *N. otitidiscaviarum*. Strikingly, three *N. farcinica* strains carrying the sulfonamide-resistant gene *sul1* exhibited elevated sulfamethoxazole/trimethoprim MIC values (>8/152 μg/mL), while *sul1* was absent in other sulfamethoxazole/trimethoprim-resistant *Nocardia* species (**Figure 4B**).

For genes conferring resistance beyond ARGs, we analyzed *warA*, *aph(2’’)*, and *gyrA* using multiple sequence alignment (**Supplementary Figure S2**). *warA*, which confers resistance to aminoglycoside antibiotics, was detected in *N. wallacei* and its related species but was absent in amikacin-resistant *N. blacklockiae*. The *aph(2’’)* gene, encoding aminoglycoside 2’’-O-phosphotransferase and mediating tobramycin resistance, was widely detected in *N. farcinica* and *N. blacklockiae*, consistent with their tobramycin-resistant phenotype; however, it was absent in other tobramycin-resistant *Nocardia* species. Ciprofloxacin resistance is potentially caused by mutations in the *gyrA* gene. In this study, we identified five amino acid substitutions in *gyrA*: Ser83Ala in *N. cyriacigeorgica*, Ser83Thr in *N. otitidiscaviarum* and *N. terpenica*, Ser83Val in *N.* sp*uti*, and both Ser83Leu and Ser83Trp in *N. wallacei*-related new species. These mutations were generally consistent with the ciprofloxacin resistance phenotype. Moreover, no *gyrA* mutation was detected in ciprofloxacin-resistant *N. farcinica* (**Supplementary Figure S2**).

The Cramér’s V analysis identified significant associations between phenotypic resistance and resistance genes. *sul1* and *qacEΔ1* were strongly associated with the sulfamethoxazole resistance phenotype (both V = 0.60, *P* < 0.001). Similarly, *bla*_AST-1_ and *bla*_FAR-1_ showed strong associations with amoxicillin resistance (V = 0.64 and 0.63, respectively; both *P* < 0.001) (**Supplementary Figure S3**). *gyrA* mutations exhibited a strong positive correlation with ciprofloxacin resistance (Cramer’s V = 0.77, *P <*0.001), while the presence of the *aph(2’’)* gene significantly correlated with tobramycin resistance (V = 0.67, *P <*0.001), and *warA* gene carriage demonstrated the strongest association with amikacin resistance (V = 0.89, *P <*0.001) (**Supplementary Figure S4**).

## 4 Discussion

This study combined species identification, AST, and WGS of clinical *Nocardia* isolates to systematically explore their phylogenetic architecture, characterize antimicrobial resistance patterns, and elucidate inter-species differences in resistance determinants genomic features with implications for both taxonomy and clinical management.

In this study, clinical *Nocardia* isolates were predominantly composed of three species: *N. farcinica* (59.5%, 88/148), *N. cyriacigeorgica* (13.5%, 20/148) and *N. otitidiscaviarum* (10.8%, 16/148), together accounting for 83.8% of all isolates. In large-scale *Nocardia* studies conducted in New Zealand (McKinney et al., 2023) and the United States (Hamdi et al., 2020), *N. nova*, *N. cyriacigeorgica*, *N. farcinica* were dominant, while in largest-scale *Nocardia* study in China (Wang et al., 2022), *N. farcinica*, *N. cyriacigeorgica*, *N. abscess*, and *N. otitidiscaviarum* were dominant, similar to this study, indicating that the prevalence trends of different species vary across regions. *N. farcinica* and *N. cyriacigeorgica* demonstrate higher clinical prevalence compared to other species in this genus.

Mass spectrometry demonstrated high accuracy in species-level identification, successfully identifying most clinically prevalent *Nocardia* species. These findings suggest its considerable potential for preliminary *Nocardia* screening in clinical settings (Carrasco et al., 2016). Consequently, primary mass spectrometric analysis of clinical isolates is an essential step in the diagnostic workflow for suspected nocardiosis. Nevertheless, it misidentified several genetically divergent isolates, particularly novel taxa, underscoring the limitations of Mass spectrometry-based approaches for complex genera. Mass spectrometry identification is based on databases, which need to be continuously improved and updated to cope with the large number of *Nocardia* species (Conville et al., 2017). ANI analysis seems to have resolved these ambiguities, leading to the reclassification of four isolates and the discovery of 14 potentially novel species, primarily within the *N. cyriacigeorgica* and *N. transvalensis* lineages. Although 130 species of *Nocardia* have been successfully named to date (https://lpsn.dsmz.de/genus/*Nocardia* ), this study shows that there are still many unnamed species of *Nocardia* in clinical practice. These findings reflect ongoing diversification within clinically relevant *Nocardia* species and underscore the need for continual taxonomic refinement based on WGS. This study suggests that the MALDI-TOF database should be updated based on the reclassification of ANI to better address the species identification of *Nocardia*.

AST revealed substantial interspecies variability in *Nocardia* species with direct implications for therapeutic strategies. Linezolid and amikacin were highly effective against the majority of clinical *Nocardia* isolates. However, their dose-dependent toxicities and adverse effects preclude their use as first-line treatments (Green et al., 2001; Sack et al., 1976). Amikacin resistance appears to be restricted to the *N. transvalensis* complex (including *N. wallacei*, *N. blacklockiae*, and *N. transvalensis*), may mediated by the *warA* mechanism, with a concordance rate of 92.2% (Hershko et al., 2024). In this study, the *warA* gene was detected exclusively in *N. wallacei* and its related species but was absent in *N. blacklockiae*. This absence suggests the potential existence of alternative resistance-conferring genes in *N. blacklockiae*, which requires further exploration.

Sulfamethoxazole/trimethoprim have been the antimicrobials of choice to treat nocardiosis (Wilson, 2012), remains the cornerstone of clinical management. The overall concordance rate between *sul1* gene and sulfamethoxazole/trimethoprim resistance was 37.5%, while the concordance rate with high MIC (>8/152 µg/mL) sulfamethoxazole/trimethoprim resistance was 100%. In our study, three *N. farcinica* strains with elevated MICs (>8/152 µg/mL) uniquely harbored the *sul1* gene, confirming *sul1*-mediated horizontal gene transfer drives high-level resistance. Previous studies on the diversity of antimicrobial resistance determinants in 76 sulfamethoxazole/trimethoprim-resistant *Nocardia* strains (MIC values ≥ 32/608 μg/mL) showed that these isolates carried one or more resistance determinants (*sul1* accounted for 93.42%, *sul2* accounted for 78.94%, and *dfrA (S1)* accounted for 14.70%) (Valdezate et al., 2015). Transposon-mediated sulfamethoxazole/trimethoprim resistance in *N. farcinica* has been elucidated in previous studies conducted in our laboratory (Che et al., 2022). In this study of *N. farcinica*, the *sul1*-mediated resistance rate was 3.4% (3/88). As the drug of choice for treating *Nocardia*, we should pay attention to the prevalence and relevance of this resistance determinant to provide effective clinical management of the resulting disease. No *sul1*, *sul2*, or *dfrA* genes were detected in other sulfamethoxazole/trimethoprim-resistant isolates (MIC values of 4/76 and 8/152 μg/mL) likely developed resistance through mutations in dihydropteroate synthase (DHPS/*folP*) and dihydropteroate reductase (DHPR/*folA*) genes (Mehta et al., 2018). Although a prior study (Hershko et al., 2023b) detected a potentially significant Ala146Val mutation in *folP2* among trimethoprim-sulfamethoxazole -resistant *Nocardia*, our analysis of *folP* and its homologs *folP2*, *folA*, *folC* (encoding dihydrofolate synthase) across 148 *Nocardia* isolates via multiple sequence alignment revealed no aberrant mutations within these genes in trimethoprim-sulfamethoxazole-resistant strains. Current understanding suggests that the precise underlying mechanisms have yet to be fully elucidated (Patamatamkul, 2025). This partial genotype-phenotype correlation highlights the complex resistance landscape for this first-line agent and warrants further molecular investigation.

*β*-lactam antibiotics are sometimes used as alternatives to sulfamethoxazole/trimethoprim for nocardiosis treatment (Wilson, 2012). Species-specific *β*-lactam resistance was evident both phenotypically and genotypically in *Nocardia* species. The overall consistency of *bla*_AST-1_ from *N.otitidiscaviarum* with all β-lactamase antibiotics was 100%, whereas in *N. cyriacigeorgica*, it was 50% for imipenem, 85% for cefepime, 100% for cefoxitin, 75% for amoxicillin/clavulanic acid, and 40% for ceftriaxone. *N.otitidiscaviarum* displayed extensive resistance to all *β*-lactam antibiotics, with universally high MIC values, consistent with previous studies (Hamdi et al., 2020). This phenotype correlated with the presence of the *bla*_AST-1_ *β*-lactamase gene homolog (74.02-74.47% nucleotide identity to class A *β*-lactamases) exclusive to this species, potentially encoding a novel broad-spectrum metallo-*β*-lactamase responsible for complete *β*-lactam resistance—a previously unreported mechanism in the *Nocardia* genus, while nucleotide identity in *N. cyriacigeorgica* was 85.52-87.54%, the *bla*_AST-1_ genes in these two *Nocardia* species may represent distinct *β*-lactamase genes. In contrast, *bla*_FAR-1_, another *β*-lactamase gene, was predominantly detected in *N. farcinica* and likely contributed to partial resistance to cephalosporins, although susceptibility to imipenem and amoxicillin/clavulanic acid remained relatively high (Laurent et al., 1999). These results emphasize the importance of distinguishing between different *β*-lactamase gene families in predicting resistance phenotypes. Our data suggest that sulfamethoxazole/trimethoprim, amikacin, and linezolid may be a reasonable empirical choice for *N. otitidiscaviarum*, though clinical validation is needed. At the same time, we must also be vigilant against the emergence of sulfamethoxazole/trimethoprim-resistant *N. otitidiscaviarum*, this is likely to lead to the limited use of antibiotics and result in death (Fu et al., 2023; Barry et al., 2022).

Tobramycin, an aminoglycoside, exhibited complete resistance in *N. farcinica* (100% non-susceptibility), this differs slightly from the 83.6% (107/128) reported in previously study (Valdezate et al., 2017) and is similar to the 99–99.5% reported by Schlaberg et al. and Hamdi et al (Schlaberg et al., 2014; Hamdi et al., 2020), whereas *N. cyriacigeorgica* and *N. otitidiscaviarum* demonstrated susceptibility rates of 90.0% and 68.8%, respectively, highlighting significant interspecies variability that precludes the empirical tobramycin use for *N. farcinica* infections. The *aph(2’’)* gene, potentially mediating tobramycin resistance (Hershko et al., 2023a), was detected exclusively in *N. farcinica* and *N. blacklockiae* in this study, showing a strong association with resistance in these species; however, it was not detected in other tobramycin-resistant *Nocardia* isolates, with a concordance rate of 92.2%. In Tobramycin-resistant *N. wallacei* and *N. nova*, there may be a potential gene similar to *aph(2’’)*. Fluoroquinolone susceptibility was similarly variable. *N. farcinica* showed high susceptibility to ciprofloxacin and moxifloxacin, whereas *N. cyriacigeorgica* and *N. otitidiscaviarum* were uniformly resistant to ciprofloxacin and minimally susceptible to moxifloxacin (Brown-Elliott et al., 2006). While a prior study (Hershko et al., 2023b) detected the Ser83Ala mutation in ciprofloxacin-resistant *Nocardia*, our investigation identified four additional mutations across distinct *Nocardia* species, all resulting from nucleotide substitutions at position 83 of the *gyrA* gene. Fluoroquinolones should be used with caution when treating *N. cyriacigeorgica* and *N. otitidiscaviarum*. These patterns highlight the need for precise species identification prior to antibiotic selection.

The universal presence of *rbpA*, *tetA(58)*, and *tetB(58)* across all isolates aligns with the widespread reduced susceptibility to rifampicin and intermediate susceptibility (>80%) to tetracyclines (e.g., doxycycline and minocycline). This resistance is likely mediated by the efflux pump systems encoded by *tetA*/*tetB(58)* (Ishikawa et al., 2004). Additionally, the *lmrC* gene, associated with lincosamide resistance, was exclusively detected in *N. cyriacigeorgica*, whereas *novA*, encoding aminocoumarin resistance, was unique to *N. otitidiscaviarum*, despite the absence of phenotypic validation. This correlation analysis revealed several consistent genotype-phenotype links, while also exposing gaps in current resistance databases, particularly in cases where resistance was observed without known genetic determinants. These findings reinforce the need to expand *Nocardia*-specific resistance gene repositories.

*sul1* and *qacEΔ1* were strongly associated with the sulfamethoxazole resistance phenotype (both V = 0.60, *P* < 0.001). Similarly, *bla*_AST-1_ and *bla*_FAR-1_ showed strong associations with amoxicillin resistance (V = 0.64 and 0.63, respectively; both *P* < 0.001). Interestingly, some resistance genes exhibited strong statistical associations (high Cramér’s V values) with resistance phenotypes that are not mechanistically related. Such discrepancies may arise from co-occurrence patterns driven by clonal spread, geographical clustering, sampling bias or other epidemiological factors rather than direct functional interactions. Cramér’s V captures the degree of association in the observed data but does not imply causality; therefore, these associations should be interpreted cautiously.

There are several strengths in this study. A major strength is its combined analysis of drug-resistant phenotypes and genotypes, elucidating the molecular basis of resistance through detailed examination of drug-resistance genes, thereby linking genotypic mechanisms to observed resistance patterns. This study further validates the utility of MALDI-TOF MS, benchmarked against ANI, as a highly accurate method for identifying clinically relevant *Nocardia* species, affirming its value for preliminary clinical screening. Additionally, it provides species-specific insights into antimicrobial resistance profiles, identifying effective antibiotics like linezolid and amikacin. Moreover, we present the first documented evidence of complete *β*-lactam resistance in *N. otitidiscaviarum* may mediated by a *bla*_AST-1_ *β*-lactamase gene homolog. This will deepen our understanding of these bacteria and enhance our ability to combat *Nocardia* infections. Our study still has several limitations. First, the diversity of strains in this study was limited, mainly focusing on *N. farcinica*, and the isolates were mainly collected from a few provinces in China. Future studies need to include a more balanced sample containing multiple clinically relevant *Nocardia* species to validate the broad applicability of these findings and explore intrageneric differences. Second, we performed antimicrobial susceptibility testing and obtained phenotypic resistance data; however, we did not carry out additional mechanistic experiments to directly link resistance phenotypes with specific resistance genes. Such experiments (e.g., gene knockout, overexpression, or transcriptomic validation) require dedicated molecular biology approaches and resources that were beyond the scope of the present study. In future work, we aim to perform these functional validations to elucidate the genetic basis of resistance and provide a more comprehensive understanding of genotype–phenotype correlations. Third, this study is the absence of follow-up clinical outcome data. Due to the prolonged treatment course required for *Nocardia* infections and the fact that all isolates were collected as baseline bacterial samples from the clinical microbiology laboratory, subsequent patient information—such as infection severity, treatment response, and prognosis—was not available. This precluded direct assessment of associations between resistance genotypes or MIC values and clinical outcomes. Future studies integrating genomic and phenotypic analyses with detailed longitudinal clinical data will be important to clarify the real-world implications of these findings. Furthermore, beyond WGS, some innovative technologies can be applied to *Nocardia* studies (Fan et al., 2024; Liu et al., 2023; Fan et al., 2025).

## 5 Conclusion

In conclusion, our study demonstrates the value of integrating phenotypic and genotypic approaches for the comprehensive characterization of *Nocardia* species. WGS proved essential not only for species delineation and novel taxon discovery but also for revealing key antimicrobial resistance determinants. The integration of genome-based diagnostics into clinical microbiology laboratories will enhance the accuracy of *Nocardia* identification and resistance prediction, ultimately guiding more effective, species-specific therapy.

## Data Availability

The raw sequencing data generated for this study have been deposited in the Genome Sequence Archive (GSA) in National Genomics Data Center under accession number CRA028987 (https://ngdc.cncb.ac.cn/gsa/browse/CRA028987), CRA011633 (https://ngdc.cncb.ac.cn/gsa/browse/CRA011633), CRA005399 (https://ngdc.cncb.ac.cn/gsa/browse/CRA005399), CRA003805 (https://ngdc.cncb.ac.cn/gsa/browse/CRA003805) (Wang et al., 2017).
