# Supplementary material for: Genomic and phenotypic characterization of antimicrobial resistance in clinical Nocardia species isolates
Source: Front Cell Infect Microbiol. 2025 Sep 18;15:1672889. doi: 10.3389/fcimb.2025.1672889 (PMC12488653; doi:10.3389/fcimb.2025.1672889)
Supplement: Supplementary file 1 [file DataSheet1.pdf]

frontiers

Supplementary Material

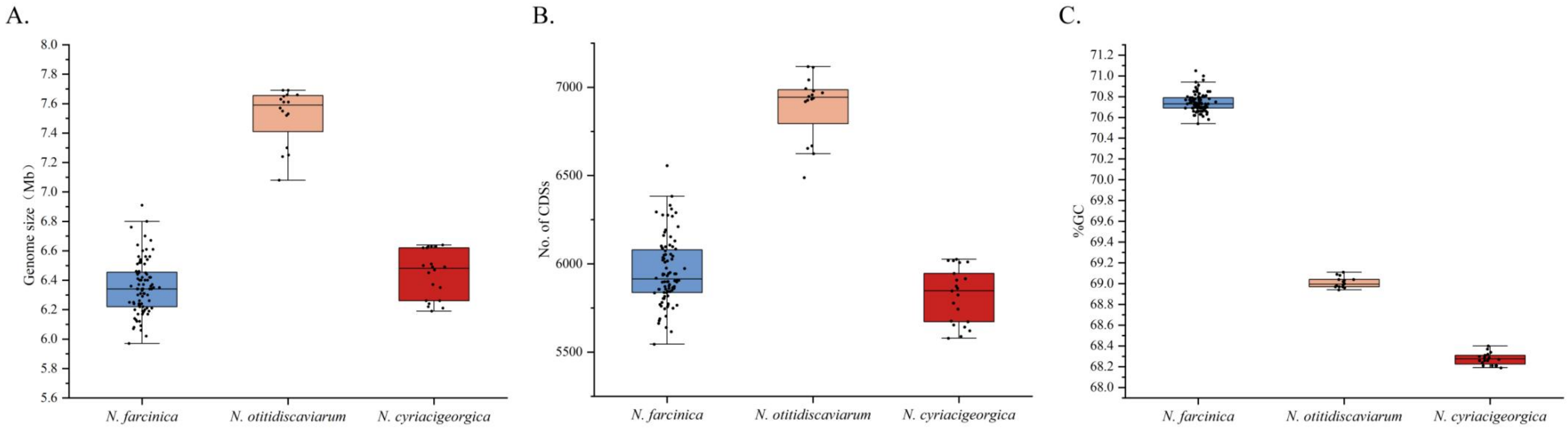

Figure S1 (A) Genome size, (B) number of coding sequences (CDSs), (C) GC content of genomes analyzed in this study.

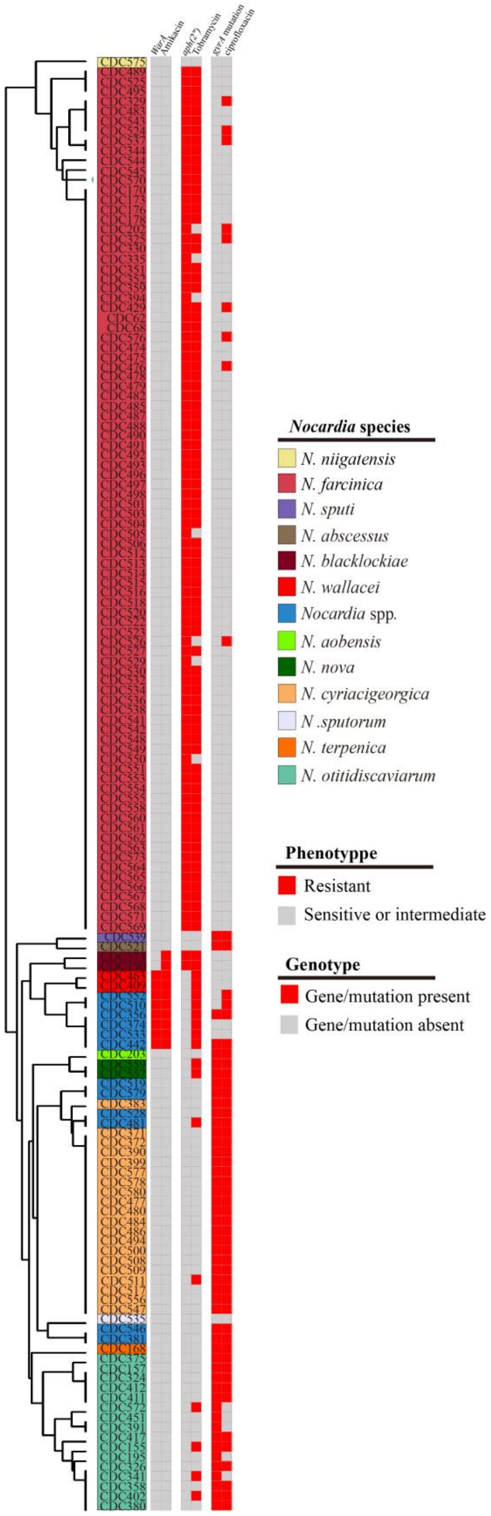

Figure S2 The presence/absence of resistance-associated genes, the occurrence of mutations, and their correlations with phenotypic resistance profiles across 148 clinical *Nocardia* isolates. *aph(2*″*)*, aminoglycoside 2″ -O-phosphotransferase gene; *WarA*. 16S rRNA m[1]A1408 methyltransferase. a: gyrA mutation including Ser83Ala, Ser83Thr, Ser83Val, Ser83Leu, Ser83Trp.

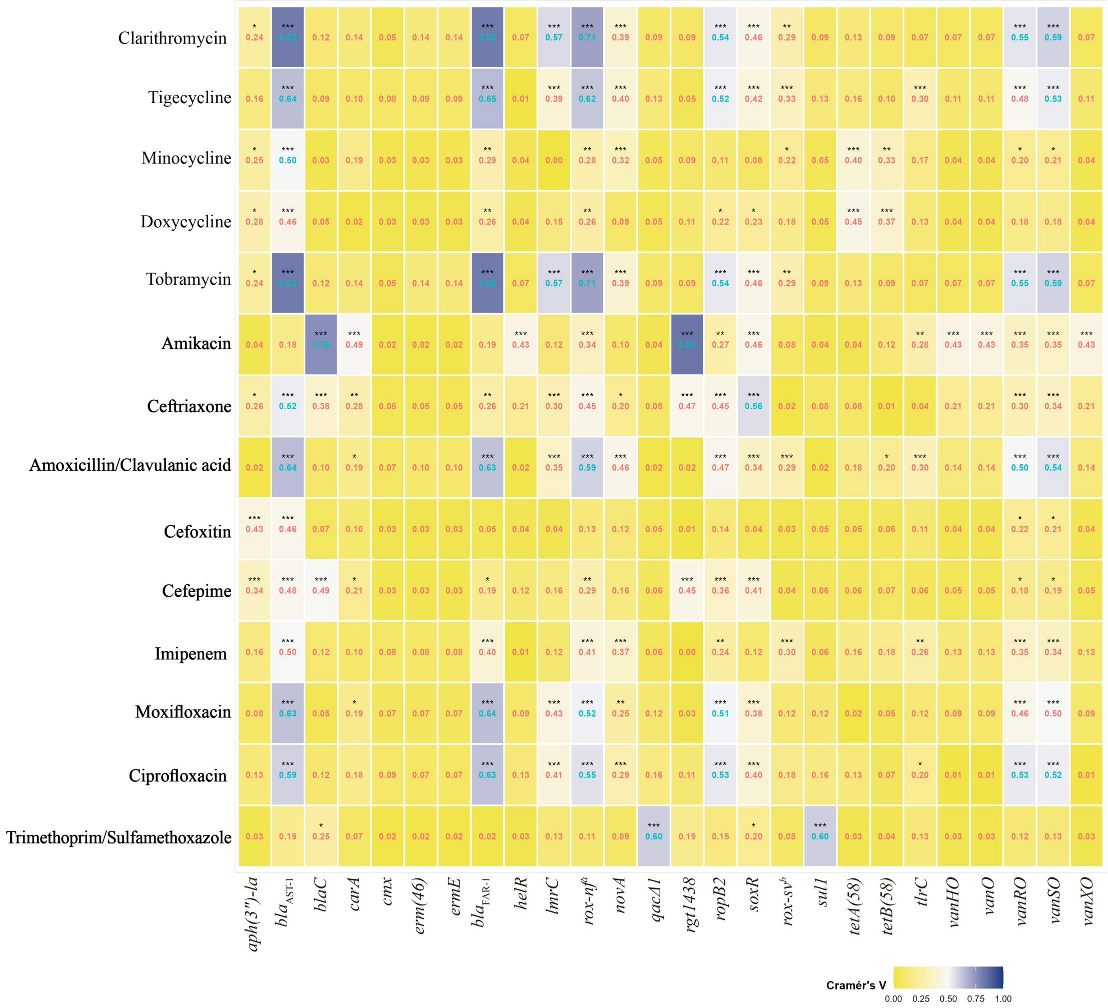

Figure S3 Cramér's V correlation analysis for phenotypic resistance associated with resistance genes. (Note: $^{*}P < 0.05$, $^{**}P < 0.01$, $^{***}P < 0.001$).

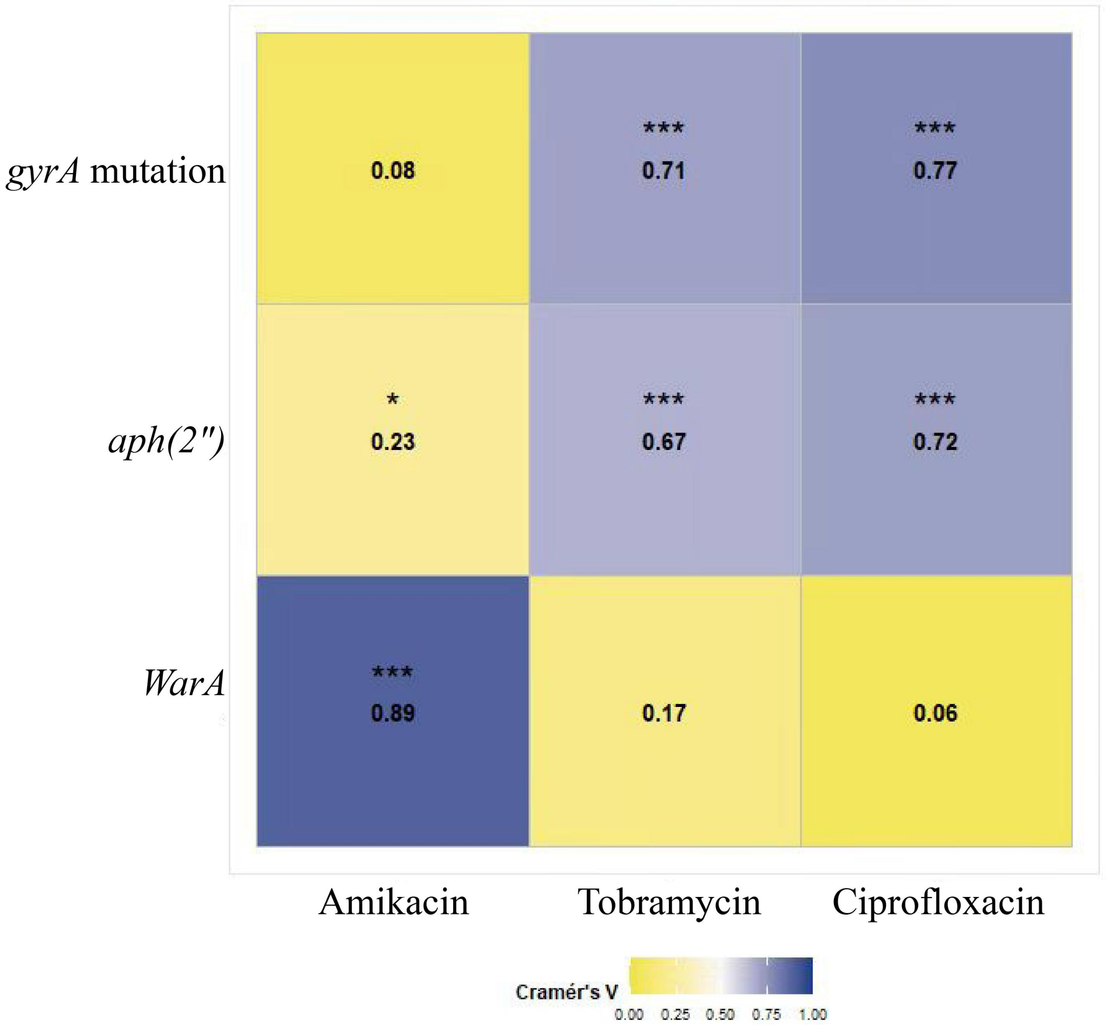

Figure S4 Cramér's V correlation analysis for the statistical association between resistance-associated genes and antimicrobial resistance phenotypes. (Note: $^{*}P < 0.05$, $^{**}P < 0.01$, $^{***}P < 0.001$).

Table S1 Matrix-Assisted Laser Desorption/Ionization Time-of-Flight Mass Spectrometry (MALDI-TOF MS) identification of clinical *Nocardia* isolates.

Strain	MALDI-TOF MS	Score	ANI result
CDC155	*N. otitidiscaviarum*	1.98	*N. otitidiscaviarum*
CDC157	*N. otitidiscaviarum*	2.15	*N. otitidiscaviarum*
CDC168	*N. terpenica*	1.74	*N. terpenica*
CDC170	*N. farcinica*	1.89	*N. farcinica*
CDC173	*N. farcinica*	2.12	*N. farcinica*
CDC176	*N. farcinica*	1.92	*N. farcinica*
CDC178	*N. farcinica*	2.22	*N. farcinica*
CDC190	*N. blacklockiae*	1.75	*N. blacklockiae*
CDC195	*N. otitidiscaviarum*	2.08	*N. otitidiscaviarum*
CDC202	*N. farcinica*	2.17	*N. farcinica*
CDC203	*N. aobensis*	2.03	*N. aobensis*
CDC324	*N. otitidiscaviarum*	1.82	*N. otitidiscaviarum*
CDC325	*N. farcinica*	2.19	*N. farcinica*
CDC326	*N. otitidiscaviarum*	1.71	*N. otitidiscaviarum*
CDC329	*N. farcinica*	2.24	*N. farcinica*
CDC330	*N. farcinica*	1.77	*N. farcinica*
CDC335	*N. farcinica*	2.01	*N. farcinica*
CDC341	*N. otitidiscaviarum*	1.85	*N. otitidiscaviarum*
CDC344	*N. farcinica*	2.11	*N. farcinica*
CDC351	*N. farcinica*	1.93	*N. farcinica*
CDC352	*N. farcinica*	2.05	*N. farcinica*
CDC356	*N. wallacei*	1.87	*Nocardia spp.*
CDC358	*N. otitidiscaviarum*	2.14	*N. otitidiscaviarum*
CDC359	*N. farcinica*	1.99	*N. farcinica*
CDC371	*N. cyriacigeorgica*	2.07	*N. cyriacigeorgica*

CDC372	*N. cyriacigeorgica*	1.79	*N. cyriacigeorgica*
CDC374	*N. wallacei*	2.16	*Nocardia spp.*
CDC375	*N. otitidiscaviarum*	1.81	*N. otitidiscaviarum*
CDC380	*N. otitidiscaviarum*	2.23	*N. otitidiscaviarum*
CDC381	*N. cyriacigeorgica*	1.76	*Nocardia spp.*
CDC383	*N. cyriacigeorgica*	2.09	*N. cyriacigeorgica*
CDC390	*N. cyriacigeorgica*	1.94	*N. cyriacigeorgica*
CDC391	*N. otitidiscaviarum*	2.13	*N. otitidiscaviarum*
CDC394	*N. farcinica*	1.84	*N. farcinica*
CDC399	*N. cyriacigeorgica*	2.02	*N. cyriacigeorgica*
CDC402	*N. otitidiscaviarum*	1.88	*N. otitidiscaviarum*
CDC409	*N. wallacei*	2.18	*N. wallacei*
CDC411	*N. otitidiscaviarum*	1.73	*N. otitidiscaviarum*
CDC412	*N. otitidiscaviarum*	2.10	*N. otitidiscaviarum*
CDC417	*N. otitidiscaviarum*	1.91	*N. otitidiscaviarum*
CDC429	*N. farcinica*	2.06	*N. farcinica*
CDC442	*N. wallacei*	1.86	*Nocardia spp.*
CDC451	*N. otitidiscaviarum*	2.20	*N. otitidiscaviarum*
CDC463	*N. wallacei*	1.78	*N. wallacei*
CDC62	*N. farcinica*	2.21	*N. farcinica*
CDC68	*N. farcinica*	1.80	*N. farcinica*
CDC574	*N. blacklockiae*	2.33	*N. blacklockiae*
CDC575	*N. niigatensis*	2.17	*N. niigatensis*
CDC576	*N. farcinica*	2.22	*N. farcinica*
CDC577	*N. cyriacigeorgica*	2.14	*N. cyriacigeorgica*
CDC578	*N. brasiliensis*	2.17	*N. cyriacigeorgica*
CDC579	*N. cyriacigeorgica*	2.01	*Nocardia spp.*
CDC580	*N. brasiliensis*	2.21	*N. cyriacigeorgica*

CDC474	*N. farcinica*	1.77	*N. farcinica*
CDC475	*N. farcinica*	2.02	*N. farcinica*
CDC476	*N. farcinica*	2.07	*N. farcinica*
CDC477	*N. cyriacigeorgica*	2.17	*N. cyriacigeorgica*
CDC478	*N. farcinica*	2.07	*N. farcinica*
CDC479	*N. farcinica*	1.85	*N. farcinica*
CDC480	*N. cyriacigeorgica*	2.21	*N. cyriacigeorgica*
CDC481	*N. cyriacigeorgica*	2.00	*Nocardia spp.*
CDC482	*N. farcinica*	1.89	*N. farcinica*
CDC483	*N. farcinica*	2.17	*N. farcinica*
CDC484	*N. cyriacigeorgica*	1.77	*N. cyriacigeorgica*
CDC485	*N. farcinica*	1.84	*N. farcinica*
CDC486	*N. cyriacigeorgica*	2.08	*N. cyriacigeorgica*
CDC487	*N. farcinica*	2.13	*N. farcinica*
CDC488	*N. farcinica*	2.03	*N. farcinica*
CDC489	*N. farcinica*	1.86	*N. farcinica*
CDC490	*N. farcinica*	2.21	*N. farcinica*
CDC491	*N. farcinica*	2.09	*N. farcinica*
CDC492	*N. farcinica*	2.22	*N. farcinica*
CDC493	*N. farcinica*	2.02	*N. farcinica*
CDC494	*N. cyriacigeorgica*	1.72	*N. cyriacigeorgica*
CDC495	*N. farcinica*	2.41	*N. farcinica*
CDC496	*N. farcinica*	2.13	*N. farcinica*
CDC497	*N. farcinica*	2.23	*N. farcinica*
CDC498	*N. farcinica*	1.87	*N. farcinica*
CDC500	*N. cyriacigeorgica*	1.8	*N. cyriacigeorgica*
CDC501	*N. farcinica*	2.08	*N. farcinica*
CDC503	*N. farcinica*	1.89	*N. farcinica*

CDC504	*N. farcinica*	2.03	*N. farcinica*
CDC505	*N. farcinica*	2.14	*N. farcinica*
CDC506	*N. farcinica*	2.02	*N. farcinica*
CDC508	*N. cyriacigeorgica*	2.2	*N. cyriacigeorgica*
CDC509	*N. cyriacigeorgica*	2.15	*N. cyriacigeorgica*
CDC510	*N. wallacei*	2.07	*Nocardia spp.*
CDC511	*N. cyriacigeorgica*	1.73	*N. cyriacigeorgica*
CDC512	*N. farcinica*	2.12	*N. farcinica*
CDC513	*N. farcinica*	1.85	*N. farcinica*
CDC514	*N. farcinica*	2.01	*N. farcinica*
CDC515	*N. farcinica*	2.12	*N. farcinica*
CDC516	*N. farcinica*	2.35	*N. farcinica*
CDC517	*N. cyriacigeorgica*	1.76	*N. cyriacigeorgica*
CDC518	*N. farcinica*	1.89	*N. farcinica*
CDC519	*N. cyriacigeorgica*	2.08	*Nocardia spp.*
CDC520	*N. farcinica*	2.11	*N. farcinica*
CDC521	*N. brasiliensis*	1.74	*N. abscessus*
CDC522	*N. farcinica*	2.20	*N. farcinica*
CDC523	*N. farcinica*	1.76	*N. farcinica*
CDC524	*N. farcinica*	2.25	*N. farcinica*
CDC525	*N. farcinica*	2.08	*N. farcinica*
CDC526	*N. farcinica*	1.81	*N. farcinica*
CDC527	*N. farcinica*	1.74	*N. farcinica*
CDC528	*N. cyriacigeorgica*	2.02	*Nocardia spp.*
CDC529	*N. farcinica*	1.73	*N. farcinica*
CDC530	*N. farcinica*	2.10	*N. farcinica*
CDC532	*N. farcinica*	2.22	*N. farcinica*
CDC533	*N. blacklockiae*	1.80	*Nocardia spp.*

CDC534	*N. farcinica*	1.87	*N. farcinica*
CDC535	*N. farcinica*	1.75	*N. sputorum*
CDC536	*N. farcinica*	2.24	*N. farcinica*
CDC537	*N. farcinica*	1.83	*N. farcinica*
CDC538	*N. farcinica*	2.23	*N. farcinica*
CDC539	*N. beijingensis*	1.78	*N. sputi*
CDC541	*N. farcinica*	2.01	*N. farcinica*
CDC542	*N. farcinica*	2.17	*N. farcinica*
CDC543	*N. farcinica*	2.13	*N. farcinica*
CDC544	*N. farcinica*	2.22	*N. farcinica*
CDC545	*N. farcinica*	1.87	*N. farcinica*
CDC546	*N. cyriacigeorgica*	1.86	*Nocardia spp.*
CDC547	*N. cyriacigeorgica*	2.08	*N. cyriacigeorgica*
CDC548	*N. farcinica*	1.72	*N. farcinica*
CDC549	*N. farcinica*	2.18	*N. farcinica*
CDC550	*N. farcinica*	1.80	*N. farcinica*
CDC551	*N. farcinica*	2.11	*N. farcinica*
CDC552	*N. wallacei*	2.15	*Nocardia spp.*
CDC553	*N. farcinica*	2.05	*N. farcinica*
CDC572	*N. otitidiscaviarum*	1.90	*N. otitidiscaviarum*
CDC554	*N. farcinica*	2.15	*N. farcinica*
CDC555	*N. farcinica*	1.85	*N. farcinica*
CDC556	*N. cyriacigeorgica*	2.20	*N. cyriacigeorgica*
CDC557	*N. nova*	2.12	*N. nova*
CDC558	*N. farcinica*	2.01	*N. farcinica*
CDC559	*N. nova*	1.80	*N. nova*
CDC560	*N. farcinica*	2.09	*N. farcinica*
CDC561	*N. farcinica*	2.14	*N. farcinica*

CDC562	*N. farcinica*	1.78	*N. farcinica*
CDC563	*N. farcinica*	2.03	*N. farcinica*
CDC573	*N. farcinica*	2.03	*N. farcinica*
CDC564	*N. farcinica*	2.00	*N. farcinica*
CDC565	*N. farcinica*	2.17	*N. farcinica*
CDC566	*N. farcinica*	2.09	*N. farcinica*
CDC567	*N. farcinica*	2.07	*N. farcinica*
CDC568	*N. farcinica*	1.87	*N. farcinica*
CDC569	*N. farcinica*	1.70	*N. farcinica*
CDC571	*N. farcinica*	2.12	*N. farcinica*
CDC570	*N. farcinica*	2.09	*N. farcinica*

Note: An identification confidence score ≥ 1.6 is reliable at the genus level, while a score ≥ 1.8 is reliable for species-level identification. Based on our phylogenomic tree and average nucleotide identity (ANI) values, four were reclassified to other species, and 12 were considered as new species.

Table S2 Classification allocation of species without a type strain in the National Center for Biotechnology Information (NCBI).

**Strain**	**Closest related type strain**	**ANI(%)**	**isDDH(%)**	**Species**
Nocardia spp. CDC159	*N. pulmonis* CDC141	99.99	98.29	*N. pulmonis*
Nocardia spp. CY15	*N. sputi* CY8	99.98	98.29	*N. sputi*
Nocardia spp. CY41	*N. beijingensis* BJ06-0111	96.42	74.60	*N. beijingensis*
Nocardia spp. MDA0666	*N. aobensis* NPDC004119	97.55	77.10	*N. aobensis*

Note: ANI, average nucleotide identity; isDDH, in silico DNA-DNA hybridization.

Table S3 General features of novel species.

**Strain**	**Closest related typestrain**	**ANI(%)**	**isDDH(%)**
CDC356	*N. wallacei* FMUON74	91.05	43.40
CDC374	*N. wallacei* FMUON74	91.02	43.40
CDC442	*N. wallacei* FMUON74	91.15	43.50
CDC533	*N. wallacei* FMUON74	90.99	43.10
CDC510	*N. wallacei* FMUON74	91.06	43.90
CDC552	*N. wallacei* FMUON74	91.12	43.80
CDC579	*N. cyriacigeorgica* MDA3349	90.79	43.20
105602	*N. cyriacigeorgica* MDA3349	90.02	40.60
CDC481	*N. cyriacigeorgica* MDA3349	90.89	43.20
CDC519	*N. cyriacigeorgica* MDA3349	90.85	43.30
CDC528	*N. cyriacigeorgica* MDA3349	90.87	43.20
CDC381	*N. cyriacigeorgica* MDA3349	90.26	40.90
CDC546	*N. cyriacigeorgica* MDA3349	90.21	40.90
AUSMDU00012717	*N. amikacinitolerans* NBRC 108937	79.39	23.10
JCM 34519	*N. amikacinitolerans* NBRC 108937	79.36	23.00

Table S4 Genome sequences of strains used in this study.

Strain	Species	Geno	%GC	No. of	References
CDC155	*N. otitidiscaviarum*	7.25	69.08	6624	This study
CDC157	*N. otitidiscaviarum*	7.61	68.97	6948	This study
CDC168	*N. terpenica*	8.80	68.54	8068	This study
CDC170	*N. farcinica*	6.08	70.81	5679	This study
CDC173	*N. farcinica*	6.37	70.69	5941	This study
CDC176	*N. farcinica*	6.13	70.74	5761	This study
CDC178	*N. farcinica*	6.36	70.69	5944	This study
CDC190	*N. blacklockiae*	8.10	69.82	7295	This study
CDC195	*N. otitidiscaviarum*	7.30	69.04	6668	This study
CDC202	*N. farcinica*	6.36	70.89	5919	This study
CDC203	*N. aobensis*	8.18	67.71	7431	This study
CDC324	*N. otitidiscaviarum*	7.66	68.97	6969	This study
CDC325	*N. farcinica*	6.37	70.69	5945	This study
CDC326	*N. otitidiscaviarum*	7.57	69.04	6919	This study
CDC329	*N. farcinica*	6.53	71.05	6074	This study
CDC330	*N. farcinica*	6.07	70.77	5663	This study
CDC335	*N. farcinica*	6.17	70.85	5757	This study
CDC341	*N. otitidiscaviarum*	7.66	69.00	6957	This study
CDC344	*N. farcinica*	6.80	70.58	6383	This study
CDC351	*N. farcinica*	6.24	70.85	5856	This study
CDC352	*N. farcinica*	5.97	70.96	5545	This study
CDC356	*Nocardia spp.*	7.73	68.87	7150	This study
CDC358	*N. otitidiscaviarum*	7.08	69.11	6488	This study
CDC359	*N. farcinica*	6.56	70.70	6154	This study

CDC371	*N. cyriacigeorgica*	6.37	68.32	5744	This study
CDC372	*N. cyriacigeorgica*	6.49	68.31	5861	This study
CDC374	*Nocardia spp.*	8.06	68.82	7521	This study
CDC375	*N. otitidiscaviarum*	7.53	68.98	6939	This study
CDC380	*N. otitidiscaviarum*	7.61	69.02	6980	This study
CDC381	*Nocardia spp.*	6.18	68.45	5672	This study
CDC383	*N. cyriacigeorgica*	6.21	68.37	5579	This study
CDC390	*N. cyriacigeorgica*	6.50	68.27	5874	This study
CDC391	*N. otitidiscaviarum*	7.63	68.97	6992	This study
CDC394	*N. farcinica*	6.21	70.85	5851	This study
CDC399	*N. cyriacigeorgica*	6.51	68.29	5916	This study
CDC402	*N. otitidiscaviarum*	7.52	68.94	6933	This study
CDC409	*N. wallacei*	7.69	69.29	7027	This study
CDC411	*N. otitidiscaviarum*	7.69	68.97	7117	This study
CDC412	*N. otitidiscaviarum*	7.69	68.96	7113	This study
CDC417	*N. otitidiscaviarum*	7.24	69.09	6654	This study
CDC429	*N. farcinica*	6.12	70.91	5782	This study
CDC442	*Nocardia spp.*	7.71	68.91	7131	This study
CDC451	*N. otitidiscaviarum*	7.65	68.99	7042	This study
CDC463	*N. wallacei*	7.70	69.24	6979	This study
CDC62	*N. farcinica*	6.34	70.73	5899	This study
CDC68	*N. farcinica*	6.18	70.94	5774	This study
CDC574	*N. blacklockiae*	8.50	69.81	7602	This study
CDC575	*N. niigatensis*	8.19	68.24	7632	This study
CDC576	*N. farcinica*	6.22	70.72	5768	This study
CDC577	*N. cyriacigeorgica*	6.35	68.19	5778	This study

CDC578	*N. cyriacigeorgica*	6.22	68.24	5642	This study
CDC579	*Nocardia spp.*	6.38	68.42	5778	This study
CDC580	*N. cyriacigeorgica*	6.26	68.26	5621	This study
CDC474	*N. farcinica*	6.61	70.70	6311	This study
CDC475	*N. farcinica*	6.46	70.85	6050	This study
CDC476	*N. farcinica*	6.26	70.79	5899	This study
CDC477	*N. cyriacigeorgica*	6.49	68.28	5847	This study
CDC478	*N. farcinica*	6.34	70.74	5900	This study
CDC479	*N. farcinica*	6.54	70.54	6270	This study
CDC480	*N. cyriacigeorgica*	6.62	68.21	6011	This study
CDC481	*Nocardia spp.*	6.39	68.42	5758	This study
CDC482	*N. farcinica*	6.34	70.75	5898	This study
CDC483	*N. farcinica*	6.22	70.87	5872	This study
CDC484	*N. cyriacigeorgica*	6.64	68.29	6026	This study
CDC485	*N. farcinica*	6.14	70.80	5689	This study
CDC486	*N. cyriacigeorgica*	6.63	68.21	6018	This study
CDC487	*N. farcinica*	6.23	70.79	5816	This study
CDC488	*N. farcinica*	6.41	70.76	6036	This study
CDC489	*N. farcinica*	6.56	70.69	6130	This study
CDC490	*N. farcinica*	6.12	70.77	5746	This study
CDC491	*N. farcinica*	6.40	70.72	6010	This study
CDC492	*N. farcinica*	6.60	70.66	6276	This study
CDC493	*N. farcinica*	6.30	70.77	5866	This study
CDC494	*N. cyriacigeorgica*	6.63	68.21	6019	This study
CDC495	*N. farcinica*	6.20	70.79	5770	This study
CDC496	*N. farcinica*	6.52	70.71	6097	This study

CDC497	*N. farcinica*	6.25	70.75	5856	This study
CDC498	*N. farcinica*	6.64	70.72	6277	This study
CDC500	*N. cyriacigeorgica*	6.62	68.21	6008	This study
CDC501	*N. farcinica*	6.24	70.69	5837	This study
CDC503	*N. farcinica*	6.46	70.63	6101	This study
CDC504	*N. farcinica*	6.29	70.63	5845	This study
CDC505	*N. farcinica*	6.70	70.62	6332	This study
CDC506	*N. farcinica*	6.40	70.73	6053	This study
CDC508	*N. cyriacigeorgica*	6.47	68.31	5908	This study
CDC509	*N. cyriacigeorgica*	6.19	68.34	5589	This study
CDC510	*Nocardia spp.*	8.25	68.78	7474	This study
CDC511	*N. cyriacigeorgica*	6.63	68.26	5946	This study
CDC512	*N. farcinica*	6.61	70.67	6211	This study
CDC513	*N. farcinica*	6.42	70.66	6084	This study
CDC514	*N. farcinica*	6.19	70.78	5819	This study
CDC515	*N. farcinica*	6.51	70.66	6107	This study
CDC516	*N. farcinica*	6.44	70.70	6022	This study
CDC517	*N. cyriacigeorgica*	6.24	68.26	5676	This study
CDC518	*N. farcinica*	6.34	70.74	5897	This study
CDC519	*Nocardia spp.*	6.60	68.38	6023	This study
CDC520	*N. farcinica*	6.42	70.73	6017	This study
CDC521	*N. abscessus*	8.20	68.29	7612	This study
CDC522	*N. farcinica*	6.45	70.70	6055	This study
CDC523	*N. farcinica*	6.23	70.81	5804	This study
CDC524	*N. farcinica*	6.35	71.00	5974	This study
CDC525	*N. farcinica*	6.52	70.61	6180	This study

CDC526	*N. farcinica*	6.19	70.70	5862	This study
CDC527	*N. farcinica*	6.21	70.66	5766	This study
CDC528	*Nocardia spp.*	6.46	68.37	5845	This study
CDC529	*N. farcinica*	6.25	70.83	5836	This study
CDC530	*N. farcinica*	6.44	70.70	6027	This study
CDC532	*N. farcinica*	6.54	70.69	6192	This study
CDC533	*Nocardia spp.*	7.91	68.82	7380	This study
CDC534	*N. farcinica*	6.40	70.80	5951	This study
CDC535	*N. sputorum*	7.93	68.83	7150	This study
CDC536	*N. farcinica*	6.35	70.73	5907	This study
CDC537	*N. farcinica*	6.76	70.73	6294	This study
CDC538	*N. farcinica*	6.54	70.74	6098	This study
CDC539	*N. sputi*	7.24	68.02	6954	This study
CDC541	*N. farcinica*	6.31	70.73	5876	This study
CDC542	*N. farcinica*	6.45	70.65	6091	This study
CDC543	*N. farcinica*	6.42	70.66	5944	This study
CDC544	*N. farcinica*	6.17	70.77	5749	This study
CDC545	*N. farcinica*	6.17	70.75	5804	This study
CDC546	*Nocardia spp.*	6.08	68.46	5533	This study
CDC547	*N. cyriacigeorgica*	6.26	68.40	5654	This study
CDC548	*N. farcinica*	6.19	70.70	5862	This study
CDC549	*N. farcinica*	6.30	70.79	5943	This study
CDC550	*N. farcinica*	6.26	70.65	5872	This study
CDC551	*N. farcinica*	6.91	70.63	6556	This study
CDC552	*Nocardia spp.*	8.08	68.75	7328	This study
CDC553	*N. farcinica*	6.51	70.72	6087	This study

CDC572	*N. otitidiscaviarum*	7.55	69.03	6927	This study
CDC554	*N. farcinica*	6.06	70.80	5640	This study
CDC555	*N. farcinica*	6.20	70.78	5843	This study
CDC556	*N. cyriacigeorgica*	6.45	68.30	5824	This study
CDC557	*N. nova*	7.85	67.91	7120	This study
CDC558	*N. farcinica*	6.56	70.66	6161	This study
CDC559	*N. nova*	7.85	67.91	7131	This study
CDC560	*N. farcinica*	6.34	70.74	5904	This study
CDC561	*N. farcinica*	6.40	70.69	6044	This study
CDC562	*N. farcinica*	6.34	70.72	5937	This study
CDC563	*N. farcinica*	6.31	70.80	5883	This study
CDC573	*N. farcinica*	6.45	70.82	6030	This study
CDC564	*N. farcinica*	6.33	70.70	5933	This study
CDC565	*N. farcinica*	6.40	70.76	5976	This study
CDC566	*N. farcinica*	6.37	70.72	5909	This study
CDC567	*N. farcinica*	6.31	70.78	5857	This study
CDC568	*N. farcinica*	6.67	70.73	6290	This study
CDC569	*N. farcinica*	6.09	70.79	5704	This study
CDC571	*N. farcinica*	6.24	70.62	5838	This study
CDC570	*N. farcinica*	6.02	70.85	5616	This study
BJ06-0146	*N. abscessus*	8.70	68.50	7647	NCBI
N-20	*N. abscessus*	7.95	68.50	7017	NCBI
NBRC 100379	*N. africana*	7.81	68.00	7071	NCBI
NCTC 13184	*N. africana*	7.75	68.00	7309	NCBI
BJ06-0157	*N. amamiensis*	8.19	67.50	7354	NCBI
NBRC 108937	*N. amikacinitolerans*	7.65	68.50	6692	NCBI

NBRC 100462	*N. anaemiae*	8.62	69.50	7604	NCBI
W9405	*N. arizonensis*	7.20	68.00	6447	NCBI
AUSMDU00012717	*Nocardia* spp.	10.01	66.50	9002	NCBI
NBRC 100137	*Nocardia* spp.	7.12	68.50	6424	NCBI
BJ06-0111	*N. beijingensis*	7.53	68.50	6647	NCBI
N-39	*N. beijingensis*	7.56	69.00	6735	NCBI
N-17	*N. blacklockiae*	6.31	70.50	5746	NCBI
ATCC 700358	*N. brasiliensis*	9.44	68.00	8414	NCBI
BJ06-0105	*N. brasiliensis*	8.61	68.00	7589	NCBI
CNM20130759	*N. cerradoensis*	9.19	67.50	8329	NCBI
CICC 11023	*N. colli*	10.03	67.50	8917	NCBI
105602	*Nocardia* spp.	6.30	68.50	5673	NCBI
BJ06-0097	*N. cyriacigeorgica*	6.73	68.00	6002	NCBI
W9944	*N. donostiensis*	5.77	66.50	4885	NCBI
X1654	*N. donostiensis*	5.80	66.50	4944	NCBI
BJ06-0114	*N. elegans*	6.51	68.00	5777	NCBI
NBRC 108235	*N. elegans*	7.54	68.00	6826	NCBI
IFM 10152	*N. farcinica*	6.29	70.50	5946	NCBI
SZ 1509	*N. farcinica*	6.61	70.50	6085	NCBI
BJ06-0141	*N. flavorosea*	6.39	67.50	5547	NCBI
120004	*N. gipuzkoensis*	8.54	68.00	7635	NCBI
234509	*N. gipuzkoensis*	8.07	68.50	7224	NCBI
BJ06-0143	*N. higoensis*	6.29	69.00	5537	NCBI
BCHNH01	*N. huaxiensis*	8.31	68.00	7401	NCBI
WCH-YHL-001	*N. huaxiensis*	8.34	67.80	7450	NCBI
CDC186	*N. implantans*	7.20	69.13	6518	NCBI

CDC192	*N. implantans*	7.21	69.00	6519	NCBI
NBRC 100128	*N. inohanensis*	8.12	68.00	7330	NCBI
NBRC 101016	*N. kruczakiae*	7.32	68.00	6595	NCBI
NBRC 108244	*N. mexicana*	8.96	68.50	8116	NCBI
NBRC 108933	*N. mikamii*	7.56	68.00	6688	NCBI
JCM 14667	*N. ninae*	9.64	67.00	8899	NCBI
NBRC 108245	*N. ninae*	9.70	67.00	8825	NCBI
NBRC 108934	*N. niwae*	7.31	68.76	6614	NCBI
ATCC 10905	*N. nova*	7.85	68.00	6923	NCBI
BAA2227	*N. nova*	7.97	68.00	7063	NCBI
BJ06 0120	*N. otitidiscaviarum*	7.55	69.00	6701	NCBI
N-22	*N. otitidiscaviarum*	7.70	69.00	6777	NCBI
NBRC 108224	*N. pseudobrasiliensis*	8.40	67.50	7863	NCBI
CDC141	*N. pulmonis*	7.86	68.50	7253	NCBI
BJ06-0122	*N. puris*	6.96	70.00	6132	NCBI
NBRC 108233	*N. puris*	7.68	70.00	6966	NCBI
NBRC 100364	*N. sienata*	6.84	68.00	6096	NCBI
CY8	*N. sputi*	7.45	68.00	6634	NCBI
CY18	*N. sputi*	8.06	68.00	7145	NCBI
IFM 12276	*N. sputorum*	7.43	68.89	6765	NCBI
IFM 12275	*N. sputorum*	7.61	69.00	6947	NCBI
AUSMDU00012715	*N. terpenica*	9.31	68.50	7855	NCBI
IFM 0406	*N. terpenica*	9.28	68.50	8144	NCBI
BJ06-0148	*N. transvalensis*	8.05	67.50	7369	NCBI
NBRC 100427	*N. vermiculata*	6.69	67.00	6062	NCBI
DSM 44445	*N. veterana*	6.80	68.00	5922	NCBI

LPB4002	*N. vulneris*	9.49	68.00	8435	NCBI
NBRC 108936	*N. vulneris*	9.38	68.00	8453	NCBI
FMUON74	*N. wallacei*	7.89	69.15	7241	NCBI
NBRC 100130	*N. yamanashiensis*	9.10	67.00	8305	NCBI
CDC153	*Nocardia* spp.	8.22	68.00	7543	NCBI
CDC159	*N. pulmonis*	7.87	68.50	7257	NCBI
CDC160	*Nocardia* spp.	8.57	67.50	7715	NCBI
CY15	*N. sputi*	8.57	67.50	7715	NCBI
CY41	*N. beijingensis*	7.86	68.50	7206	NCBI
JCM 34519	*Nocardia* spp.	9.39	67.00	8617	NCBI
JMUB6875	*Nocardia* spp.	8.34	68.00	7659	NCBI
MDA0666	*N. aobensis*	7.02	68.00	6197	NCBI

Note: CDSs, coding sequence.

frontiers

Table S5 Antimicrobial resistance profiles of other Nocardia species to 15 antibiotics.

Species / S / I / R	*N. niigatensis*	*N. puti*	*N. sputorum*	*N. wallacei*	*N. abscessus*	*N. aobensis*	*N. blacklockiae*	*N. nova*	*N. terpenica*	*Nocardia* spp.
TMP-SMX	1 / 0 / 0	1 / 0 / 0	1 / 0 / 0	2 / 0 / 0	1 / 0 / 0	1 / 0 / 0	2 / 0 / 0	2 / 0 / 0	1 / 0 / 0	10 / 0 / 2
LZD	1 / 0 / 0	1 / 0 / 0	1 / 0 / 0	2 / 0 / 0	1 / 0 / 0	1 / 0 / 0	2 / 0 / 0	2 / 0 / 0	1 / 0 / 0	12 / 0 / 0
CIP	1 / 0 / 0	0 / 0 / 1	0 / 1 / 0	1 / 1 / 0	0 / 0 / 1	0 / 0 / 1	2 / 0 / 0	0 / 0 / 2	0 / 0 / 1	1 / 1 / 10
IMP	0 / 0 / 1	0 / 1 / 0	0 / 0 / 1	0 / 0 / 2	0 / 0 / 1	1 / 0 / 0	1 / 0 / 1	2 / 0 / 0	1 / 0 / 0	7 / 4 / 1
MFX	1 / 0 / 0	0 / 0 / 1	1 / 0 / 0	2 / 0 / 0	0 / 0 / 1	0 / 0 / 1	2 / 0 / 0	0 / 0 / 2	1 / 0 / 0	3 / 5 / 4
FEP	0 / 0 / 1	1 / 0 / 0	1 / 0 / 0	0 / 0 / 2	1 / 0 / 0	1 / 0 / 0	1 / 1 / 0	2 / 0 / 0	0 / 1 / 0	10 / 2/ 0
FOX	0 / 1 / 0	1 / 0 / 0	1 / 0 / 0	0 / 0 / 2	1 / 0 / 0	0 / 1 / 0	0 / 0 / 2	1 / 0 / 1	0 / 0 / 1	0 / 5 / 7
AMC	0 / 1 / 0	0 / 0 / 1	1 / 0 / 0	0 / 1 / 1	1 / 0 / 0	0 / 0 / 1	1 / 1 / 0	1 / 0 / 1	0 / 0 / 1	6 / 2 / 4
AMK	1 / 0 / 0	1 / 0 / 0	1 / 0 / 0	0 / 0 / 2	1 / 0 / 0	1 / 0 / 0	0 / 0 / 2	2 / 0 / 0	1 / 0 / 0	6 / 0 / 6
CRO	0 / 1 / 0	1 / 0 / 0	1 / 0 / 0	2 / 0 / 0	1 / 0 / 0	1 / 0 / 0	2 / 0 / 0	1 / 1 / 0	0 / 1 / 0	12 / 0 / 0
DOX	0 / 1 / 0	1 / 0 / 0	0 / 1 / 0	0 / 0 / 2	1 / 0 / 0	0 / 0 / 1	0 / 1 / 1	0 / 2 / 0	0 / 0 / 1	1 / 11 / 0
MIN	0 / 1 / 0	1 / 0 / 0	0 / 1 / 0	0 / 1 / 1	1 / 0 / 0	0 / 1 / 0	0 / 2 / 0	0 / 2 / 0	0 / 0 / 1	1 / 10 / 1
TGC	1 / 0 / 0	1 / 0 / 0	1 / 0 / 0	0 / 0 / 2	1 / 0 / 0	0 / 0 / 1	1 / 0 / 1	1 / 0 / 1	1 / 0 / 0	8 / 0 / 4
TOB	1 / 0 / 0	1 / 0 / 0	1 / 0 / 0	0 / 0 / 2	1 / 0 / 0	0 / 0 / 1	0 / 0 / 2	0 / 0 / 2	1 / 0 / 0	4 / 1 / 7
CLR	1 / 0 / 0	0 / 1 / 0	1 / 0 / 0	0 / 0 / 2	0 / 1 / 0	0 / 0 / 1	2 / 0 / 0	2 / 0 / 0	0 / 0 / 1	0 / 3 / 9

Note: S: Susceptible, I: Intermediate, R: Resistance; AMK: Amikacin, AMC: Amoxicillin/Clavulanic acid, FEP: Cefepime, FOX: Cefoxitin, CRO: Ceftriaxone, CIP:Ciprofloxacin, CLR: Clarithromycin, DOX: Doxycycline, IMP: Imipenem, LZD: Linezolid, MIN: Minocycline, MFX: Moxifloxacin, TGC: Tigecycline, TOB: Tobramycin, TMP-SMX: Trimethoprim/Sulfamethoxazole.

Table S6 Antimicrobial susceptibility to trimethoprim/sulfamethoxazole was determined using Minimum inhibitory concentration (MIC) test strips.

**Strain**	**Figure**	**MIC(mg/L)**
CDC489	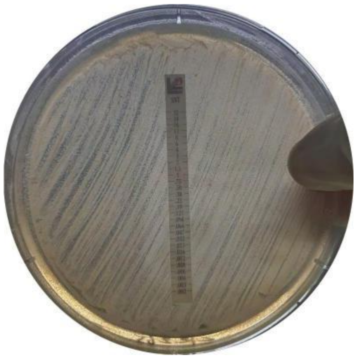	>32
CDC494	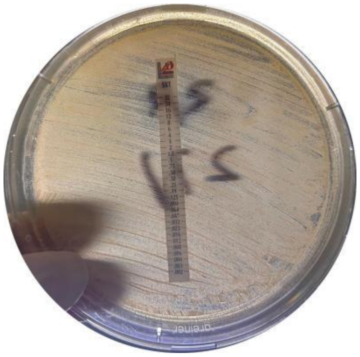	>32
CDC495	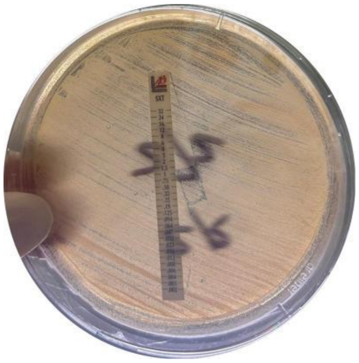	>32

CDC500	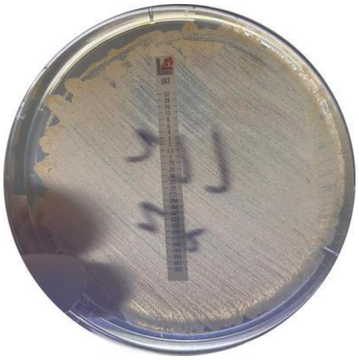	>32
CDC510	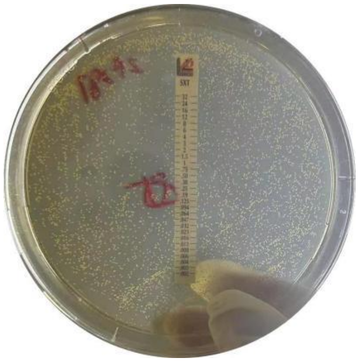	>32
CDC517	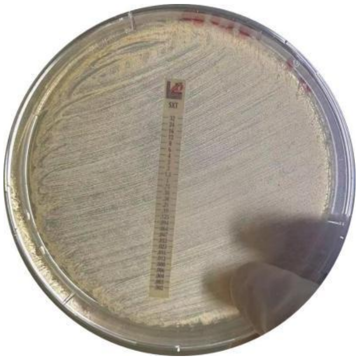	>32
CDC525	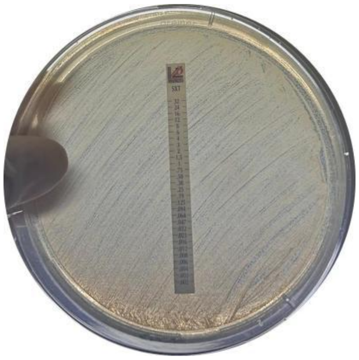	>32

CDC552	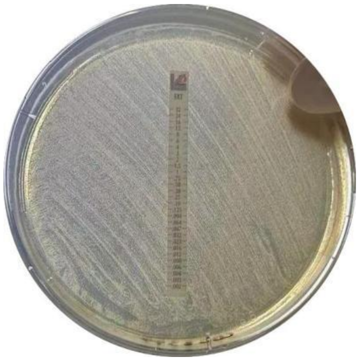	>32

Note: MIC, Minimum Inhibitory Concentration.
